# A Case of a 50-Year-Old Woman with Typical Fabry Disease Who Showed Serial Electrocardiographic and Echocardiographic Changes over a 17-Year Period

**DOI:** 10.1155/2019/9385361

**Published:** 2019-04-01

**Authors:** Su Nam Lee, Gee-Hee Kim, Ki-Dong Yoo

**Affiliations:** Division of Cardiology, Department of Internal Medicine, St. Vincent's Hospital, The Catholic University of Korea, Seoul, Republic of Korea

## Abstract

Fabry disease (FD) is a progressive, X-linked lysosomal storage disorder caused by a deficiency of *α*-galactosidase A activity. Affected individuals accumulate globotriaosylceramide and glycosphingolipids in the lysosomes and cytoplasm of cells throughout the body, leading to major organ failure and premature death. Cardiac involvement includes left ventricular hypertrophy, arrhythmia, endothelial dysfunction at vascular wall, and cardiomyopathy. The diagnosis of FD can be difficult and there is often a long lag time between symptoms and diagnosis. Here, we present a case of a 50-year-old woman with typical Fabry disease who showed serial electrocardiographic and echocardiographic changes over 17 years prior to diagnosis with Fabry disease.

## 1. Introduction

Fabry disease (FD) is a progressive inherited metabolic disorder. Deficient activity of lysosomal *α*-galactosidase A (*α*-GLA) results in progressive accumulation of globotriaosylceramide (Gb3) and related glycosphingolipids within lysosomes and, ultimately, leads to multiorgan dysfunction of the cardiac, renal, and cerebrovascular systems [1, 2]. Life- threatening cardiovascular or cerebrovascular complications limit life expectancy [3]. Therefore, early diagnosis of FD before cardiocerebrovascular irreversible organ damage occurs is important. However, the diagnosis of FD is difficult and is made at approximately 13.7 and 16.3 years in males and females, respectively, after the onset of symptoms [4]. Cardiac involvement includes left ventricular hypertrophy (LVH), arrhythmia, angina, and dyspnea. Electrocardiographic (ECG) changes in patients with FD are frequent and include LVH, ST segment depression, T wave inversion, short PR interval, prolonged QTc intervals, intermittent supraventricular tachycardia, ventricular tachycardia, atrioventricular (AV) node blocks, and bundle branch blocks [5, 6].

Here, we report a case of a 50-year-old woman who showed serial electrocardiographic and echocardiographic changes over 17 years prior to diagnosis with typical FD.

## 2. Case Presentation

**Figure 1:**
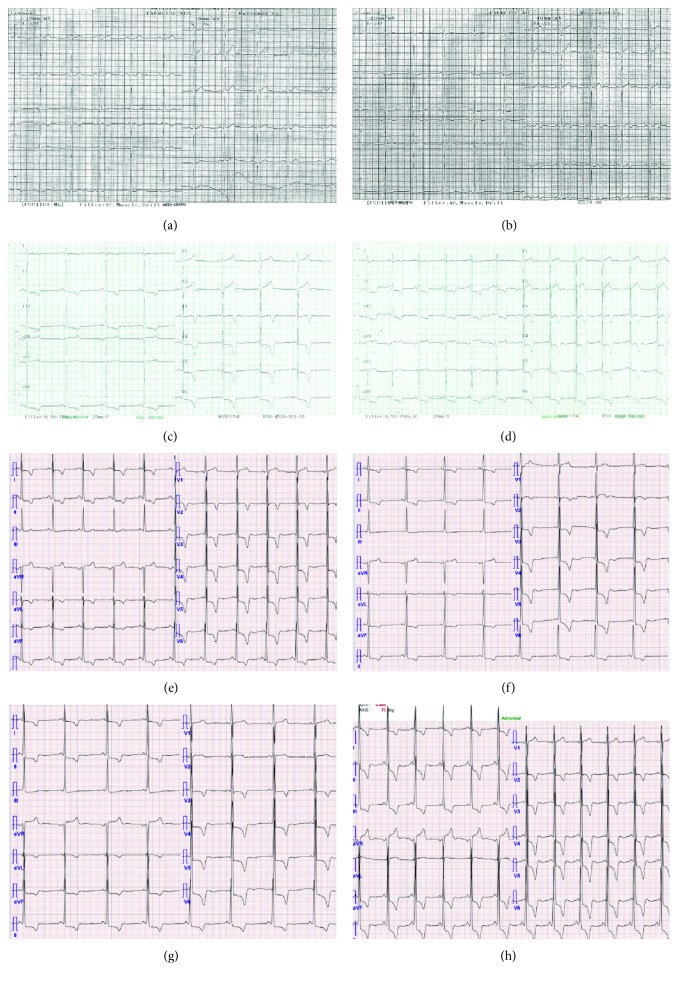
Serial electrocardiographic changes. (a) In 2000: PR interval 160 ms, LVH criteria (Sokolow-Lyon index) 28 mm. (b) In 2003: PR interval 160 ms, LVH criteria (Sokolow-Lyon index) 36 mm. (c) In 2007: PR interval 160 ms, LVH criteria (Sokolow-Lyon index) 38 mm. (d) In 2008: PR interval 120 ms, LVH criteria (Sokolow-Lyon index) 47 mm. (e) In 2010: PR interval 120 ms, LVH criteria (Sokolow-Lyon index) 46 mm. (f) In 2014: PR interval 100 ms, LVH criteria (Sokolow-Lyon index) 50 mm. (g) In 2016: PR interval 100 ms, LVH criteria (Sokolow-Lyon index) 63 mm. (h) In 2017: PR interval 100 ms, LVH criteria (Sokolow-Lyon index) 67 mm.

In 2000, a 34-year-old woman without disease was referred due to epigastric discomfort. A physical examination revealed no abnormal findings. Endoscopic examination showed normal findings. An electrocardiogram (ECG) showed regular sinus rhythm with a normal PR interval (160 ms) and no LVH by the Sokolow-Lyon index (28 mm) (Figure 1(a)). The Sokolow-Lyon index for LVH defined as *S* in V1+R in V5 or V6 (whichever is larger) ≥ 35 mm [7].

The patient was repeatedly admitted to our hospital from 2003 to 2010 (Table 1).

**Figure 2:**
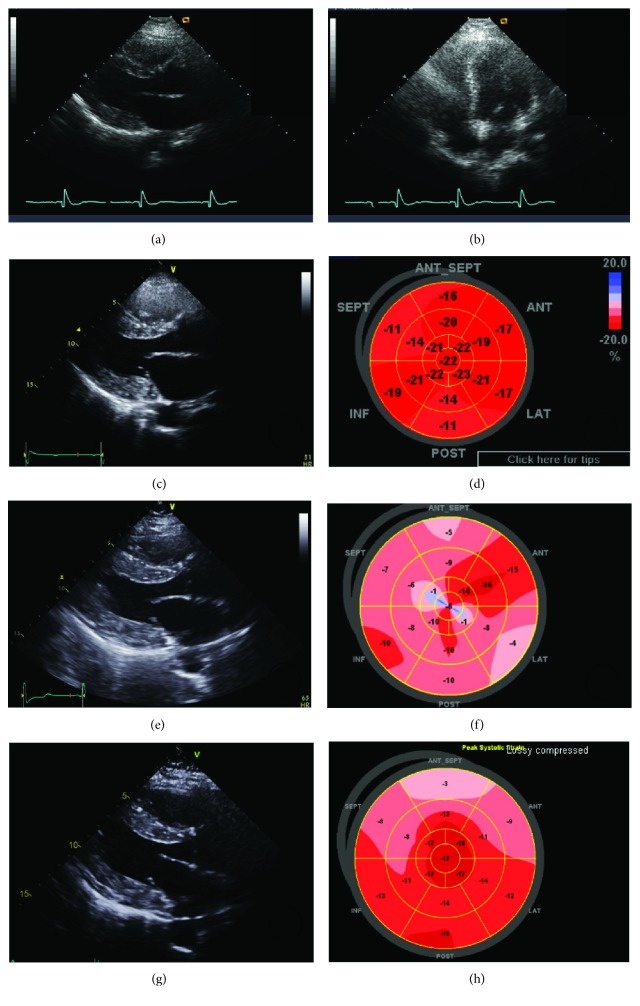
Transthoracic echocardiogram. Echocardiography showed concentric LVH in 2010 (a, b). In 2014, transthoracic echocardiography (TTE) revealed LVH (c) and partially decreased left ventricular (LV) global longitudinal strain rates (d). In 2016, TTE showed LVH (e) and marked decreased LV longitudinal strain rates (f). Eight months after ERT, TTE still showed LVH (g) but a marked improvement of LV longitudinal strain rates (h).

In 2014, the patient was referred to our hospital with dyspnea and chest pain. An ECG showed a shorter PR interval (100 ms) and more severe LVH (50 mm) by the Sokolow-Lyon index than the previous examinations (Figure 1(f)). Laboratory testing revealed a normal creatine phosphokinase (CPK) level (132 U/L; normal range 60-190 U/L), an elevated creatine kinase- (CK-) MB isoenzyme level of 15.44 ng/mL (normal range 0.1-6.7 ng/mL), and a slightly elevated lactate dehydrogenase (LDH) level of 302 U/L (normal range 140-271 U/L). TTE revealed LVH and partially decreased LV global longitudinal strain rates (Figures 2(c) and 2(d)).

**Figure 3:**
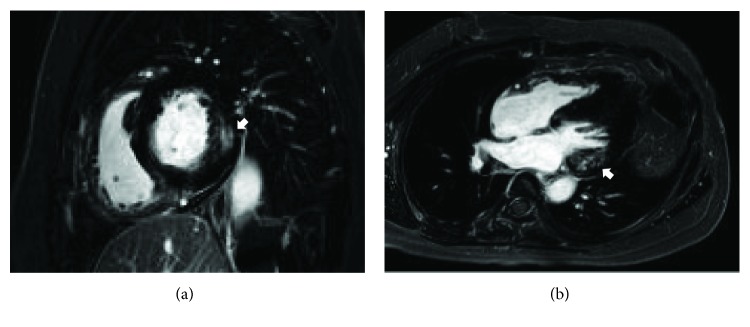
Magnetic resonance imaging. Magnetic resonance imaging showed delayed enhancement (arrows) at the basal segment of LV lateral wall. (a) Short axis view. (b) 4-chamber view.

**Figure 4:**
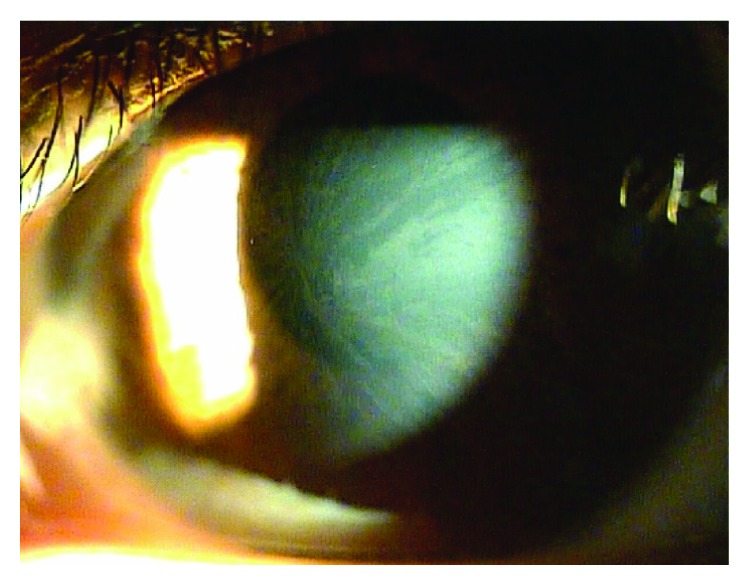
Cornea. Subepithelial lines show the typical pattern of the so-called “cornea verticillata”.

In 2016, the patient was again hospitalized with chest discomfort. The blood pressure was normal. An ECG showed a short PR interval (100 ms) and severe LVH (63 mm) by the Sokolow-Lyon index (Figure 1(g)). Laboratory testing revealed elevated CK-MB (15.21 ng/mL; normal range 0.1-6.7 ng/mL), LDH (494 U/L; normal range 140-271 U/L), and brain natriuretic peptide (pro-BNP) levels (2223 pg/mL; normal range < 115 pg/mL) with a normal CPK level of 151 U/L (normal range 60-190 U/L). Creatinine was normal and the 24-hour creatinine clearance ratio (Ccr) was 101.1 mL/min/1.73m^2^. TTE showed a thicker LV wall than the previous results. In addition, TTE revealed marked decreased LV longitudinal strain rates (Figures 2(e) and 2(f)). Magnetic resonance imaging findings showed a delayed enhancement at the basal segment of LV lateral wall and LVH, and thus, FD was suspected (Figures 3(a) and 3(b)). Ophthalmic examination showed cornea verticillata (Figure 4). We measured *α*-GLA in patient's blood plasma using a fluorometric enzyme assay. Leukocyte *α*-GLA was significantly reduced to 10.6 nmol/h/mg protein (normal range > 35 nmol/h/mg protein). We identified one hemizygous mutation in exon 6 of GLA, c.969delC (p.Leu324 Trpfs^∗^24). Therefore, this patient was diagnosed with classic FD.

**Figure 5:**
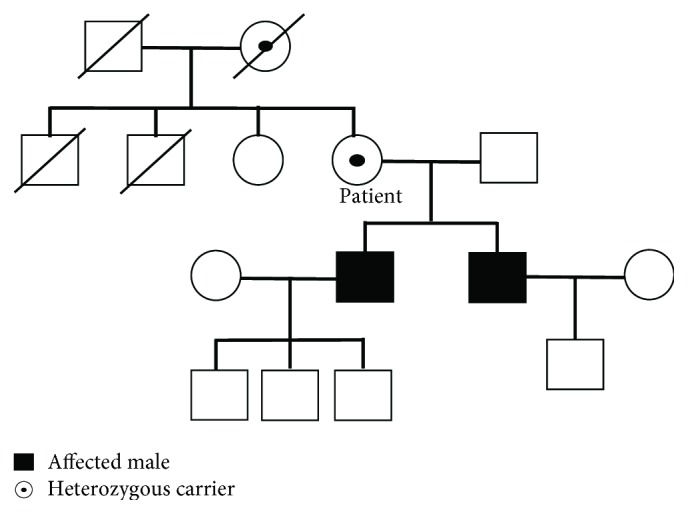
The pedigree of the patient's family.

In 2017, the patient was admitted to cardiology for enzyme replacement therapy (ERT). Before ERT, an ECG still showed a short PR interval (100 ms) and extreme LVH (67 mm) by the Sokolow-Lyon index (Figure 1(h)). Laboratory testing revealed elevated CK-MB/CPK (38.91 ng/mL; normal range 0.1-6.7 ng/mL/272 U/L; normal range 60-190 U/L), LDH (289 U/L; normal range 140-271 U/L), and pro-BNP level (2853 pg/mL; normal range < 115 pg/mL). Eight months after ERT, ECG still showed a short PR interval and extreme LVH. TTE still showed LVH but a marked improvement of LV longitudinal strain rates (Figures 2(g) and 2(h)). The patient's family members also underwent genetic testing, and the patient's two sons were diagnosed with classic FD (Figure 5). We summarized the organ involvement and changes of Gb3 and lysoGb3 biomarkers of the affected family member in Tables 2 and 3.

## 3. Discussion

FD was identified in 1898 by Anderson and Fabry [8]. This inborn error of metabolism is characterized by either an absence or deficiency of *α*-GLA activity. The enzyme substrate, Gb-3, accumulates in a variety of cell types, including capillary endothelial, renal, cardiac, and nerve cells. The first clinical symptoms occur during childhood or adolescence and include a burning pain originating in the extremities, fever of unknown origin, hypohidrosis, and gastrointestinal symptoms, such as abdominal cramps and diarrhea [9]. More specific manifestations, such as angiokeratoma and asymptomatic corneal opacities, usually present in late adolescence. Neurological, cardiac, and renal complications develop in the third or fourth decade [2].

Early signs of cardiac involvement may be present during adolescence. These signs include a shortened PR interval, arrhythmias, impaired heart rate variability, and mild valvular insufficiency [10]. Cardiac symptoms including LVH, arrhythmia, angina, and dyspnea are reported in approximately 40-60% of patients with FD [5, 6]. With an ECG, these early-stage patients had shorter PQ intervals due to a shortening of the P-wave duration as compared to age- and heart rate-matched healthy controls [11, 12]. LVH most commonly manifests at an average age of 32 years in men and 40 years in women [13].

Progressive myocardial fibrosis develops with both interstitial and replacement fibrosis as the patient ages [14]. In end-stage patients, transmural replacement fibrosis gradually reduces cardiac function to the stage of congestive heart failure [13, 15]. Therefore, early diagnosis of FD is important, although the diagnosis of FD is difficult and there is often a long lag time between symptoms and diagnosis. Males are diagnosed at a median age of 24 years old and females at a median age of 31 years old. Diagnoses are made after 15 years for males and 18 years for females after the onset of symptoms [16].

In this case, changes in ECG had been observed in the patient's late 30s, specifically a shortened PR interval and severe LVH. We should have tested for FD in this patient with unexplained LVH. However, we did not consider FD at that time. Unlike in females, ECG changes in males occur in the early 30s because the activity of enzyme is lower than that in female. Gender differences are one of the reasons why it is difficult to diagnose FD. In female patients, the progression toward hypertrophy is prolonged, whereas the development of fibrosis and regional functional abnormalities progresses simultaneously. However, in males, LVH and concomitant reduction in longitudinal function appear in adolescence and both two processes lead to replacement fibrosis [17, 18].

## 4. Conclusion

This case has not been reported previously, showing a series of long-term electrocardiographic changes prior to FD diagnosis. We reported here that serial ECG and TTE changes over 17 years were observed before the patient was diagnosed with FD. Physicians should be aware of the importance of electrocardiographic and echocardiographic changes, especially PR interval and LVH, in the diagnosis of FD.

## Figures and Tables

**Table 1:** Hospitalization history between 2003 and 2010.

Year	Age	Symptoms	Evaluation
2003	37	Palpitation and chest discomfort	ECG: a normal PR interval (160 ms) and LVH (36 mm) by the Sokolow criteria with T wave inversion on V4-6 (Figure 1(b)) Thyroid function test: normal Transthoracic echocardiography (TTE): concentric LVH Treadmill test: normal 24-hour Holter monitoring: normal

2007	41	Palpitation and chest discomfort	ECG: LVH (38 mm) by the Sokolow-Lyon index but a normal PR interval (160 ms) (Figure 1(c)) 24-hour Holter monitoring: normal Myocardial stress single-photon emission computed tomography: normal

2008	42	Palpitation and chest discomfort	ECG: a short PR interval (120 ms) and marked LVH (47 mm) by the Sokolow-Lyon index (Figure 1(d)) Coronary angiography: myocardial bridging in the mid portion of the left anterior descending artery

2010	44	Palpitation and chest discomfort	ECG: a short PR interval (120 ms) and marked LVH (46 ms) by the Sokolow-Lyon index (Figure 1(e)) TTE: concentric LVH (Figures 2(a) and 2(b))

^∗^ECG: electrocardiography; LVH: left ventricular hypertrophy.

**Table 2:** Organ involvements of the affected family member.

Variables	Patient	First son	Second son
Age at diagnosis	50	32	31
Sex	Female	Male	Male
Mutation	Exon 6 p.Leu324Trpfs^∗^24	Exon 6 p.Leu324Trpfs^∗^24	Exon 6 p.Leu324Trpfs^∗^24
Leukocyte *α*-galactosidase (nmol/h/mg protein)	10.6	2.2	4.4
PR interval on ECG	0.127	0.141	0.157
LVH on ECG	(+)	(+)	(+)
TTE	LVH	LVH	LVH
Delayed enhancement on heart MRI	(+), basal segment	(-)	(-)
Proteinuria (mg/24 h)	60	473	416
Cornea verticillata	(+)	(+)	(+)
Angiokeratoma	(+)	(+)	(+)
Anhidrosis	(-)	(-)	(+)
Chronic neurotic pain	(+)	(+)	(+)
Brain involvement	(-)	(-)	(-)

^∗^ECG: electrocardiography; TTE: transthoracic echocardiography; LVH: left ventricular hypertrophy; MRI: magnetic resonance imaging.

**Table 3:** The changes of Gb3 and lysoGb3 biomarkers of the affected family member.

	Base	3 months	6 months	9 months	12 months
Gb3 (*μ*g/mL, normal range 3.9-9.9 *μ*g/mL)					
Patient	7.4	5.3	6.5	7	7.3
First son	14.1	8.8	6.3	7.1	7.7
Second son	8.5	7.7	6.4	7.6	5.8
LysoGb3 (ng/mL, normal range ≤ 1.74 ng/mL)					
Patient	Not checked	7.82	8.13	7.14	8.05
First son	Not checked	30.4	20.8	24.3	30.8
Second son	Not checked	36	25.7	32.5	28

^∗^Gb3: globotriaosylceramide.
